# Microbiological Effectiveness of Sodium Hypochlorite Gel and Aqueous Solution When Implemented for Root Canal Disinfection in Multirooted Teeth: A Randomized Clinical Study

**DOI:** 10.3390/jfb14050240

**Published:** 2023-04-24

**Authors:** Niral Kotecha, Nimisha Chinmay Shah, Rohan Jiteshkumar Doshi, Karkala Venkappa Kishan, Alexander Maniangat Luke, Krishna Prasad Shetty, Mohammed Mustafa, Ajinkya M. Pawar

**Affiliations:** 1 Department of Conservative Dentistry and Endodontics, K M Shah Dental College and Hospital Sumandeep Vidyapeeth, Pipariya, Waghodia, Vadodara 391760, Gujarat, India; 2 Department of Clinical Science, College of Dentistry, Ajman University, Al-Jurf, Ajman 346, United Arab Emirates; 3 Center of Medical and Bio-allied Health Sciences Research, Ajman University, Al-Jurf, Ajman 346, United Arab Emirates; 4 Department of Conservative Dental Sciences, College of Dentistry, Prince Sattam Bin Abdulaziz University, P.O. Box 173, Al-Kharj 11942, Saudi Arabia; 5 Centre for Transdisciplinary Research, Department of Conservative Dentistry and Endodontics, Saveetha Dental College, Saveetha University, Chennai 600077, Tamil Nadu, India; 6 Department of Conservative Dentistry and Endodontics, Nair Hospital Dental College, Mumbai 400008, Maharashtra, India

**Keywords:** colony-forming units, disinfection, root canal therapy, sodium hypochlorite

## Abstract

The aim of endodontic therapy is to use various antimicrobial medications for proper cleaning and shaping to create an environment free of microorganisms by eradicating as many as possible from the root canal space. Even although it is a gold standard irrigant, sodium hypochlorite (NaOCl) is known for its cytotoxic effects on vital periapical tissues, making its higher concentrations inappropriate for use in conditions such as wide, underdeveloped, or damaged apices and in cases of perforations. Consequently, if it is ascertained that a gel form of sodium hypochlorite has equivalent antibacterial activity to the aqueous solution form, it could be employed in such situations. The aim of this study was the microbiologic evaluation of 5.25% sodium hypochlorite gel and aqueous solution as root canal disinfectants in multirooted teeth with primary endodontic lesions. Following ethical approval and CTRI registration, 42 patients who gave their consent and had multirooted teeth with pulpal necrosis and asymptomatic apical periodontitis were considered for the study. Following the opening of the access, pre-endodontic build up in case of class-II cavities and working length determination, a pre-operative sample (S1), which was regarded as the pre-operative microbial load of that canal, was acquired from the largest canal using a sterile paper point while maintaining strict isolation and disinfection. The computer randomization approach was used to divide the teeth into two groups at random just before beginning of chemo-mechanical preparation: Group A (*n* = 21)—canal disinfection with 5.25% sodium hypochlorite gel; Group B (*n* = 21)—canal disinfection with 5.25% sodium hypochlorite aqueous solution. Following the canal disinfection, a post-operative (S2) sample which was regarded as the postoperative microbial load of that canal was collected from the same canal using a sterile paper point. The Colony-Forming Units (CFUs) for the S1 and S2 samples were determined after 48 h aerobic incubation on Brain Heart Infusion (BHI) agar plates. The patients and the microbiologist were blinded throughout the procedure. Using SPSS 20.0 software (USA), the Shapiro–Wilk test and the Lilliefors Significance Correction were used for normality, followed by the Mann–Whitney U test which was used to compare the CFU difference (×10^5^) between the two groups. A *p* value of <0.05 was perceived as statistically significant. The mean colony-forming units count difference between the 5.25% sodium hypochlorite gel and aqueous solution groups did not differ in a manner that was statistically significant (*p* = 0.744). In multirooted teeth with primary endodontic lesions, the 5.25% sodium hypochlorite gel and the aqueous solution demonstrated comparable antimicrobial effectiveness when implemented as root canal disinfectants.

## 1. Introduction

The oral cavity is home to more than 700 species of microorganisms from 11 divisions, and these microbes are major contributors to pulpal and periapical infections [1].

The bacteria associated with primary endodontic infection are mixed, but are predominantly gram-negative anaerobic rods, whereas the bacteria associated with secondary infection comprise only one or a few bacterial species that have a tendency to form biofilms [1,2]. Bacteria existing in the biofilm show high degrees of resistance to antimicrobial agents because of the structure and physiology of the biofilm [2]. Therefore, clinical studies have demonstrated that the bacterial biofilm may remain even after thorough mechanical and chemical preparation of the root canal system.

The prime objective of endodontic therapy is to employ various antimicrobials for optimum cleaning and shaping to eliminate as many of these microbes from the root canal space as possible in order to create an environment free of them [2,3].

Regrettably, it is regularly challenging to completely eradicate microbes from root canals using instrumentation alone due to the structure of the root canal system, supplementary root canals, apical ramifications, and bacterial penetration deep into the dentinal tubules. Antimicrobial solution irrigation is thought to be important in this situation [4]. Among these antimicrobial solutions, sodium hypochlorite is widely regarded as the gold standard due to its ability, in all concentrations between 0.5 and 5.25%, to dissolve tissue, lubricate, and fight against microorganisms such as E. faecalis [5].

However, if sodium hypochlorite is inadvertently infused into the periapical region, it can have severe side effects such haemolysis, necrosis, inflammatory response, and tissue destruction. This is also the reason why clinicians are reluctant to use higher concentrations of sodium hypochlorite, especially in scenarios such as immature apex or root resorptions or perforations, which will in turn compromise the disinfection of root canals [6]. As a result, it is suggested that the gel form of sodium hypochlorite be employed in the same concentration to tackle these issues. It is composed of sodium hypochlorite (5.25% of active chlorine), a surfactant for its stability, and a gel base for increased viscosity which results in its lesser tendency to apical extrusion, thus making it useful in teeth with wide, underdeveloped, or damaged apices and in cases of perforations [7]. As a consequence, it is anticipated that utilizing sodium hypochlorite gel will lessen apical extrusion of debris and associated negative effects.

Passive ultrasonic irrigation is another tool for effectively disinfecting the root canal space. It is known to eliminate biofilms created by various bacteria via greater penetration of the antimicrobial irrigant into dentinal tubules by using an ultrasonically activated file or smooth wire within the root canal space immediately following the completion of canal preparation. Thus, its use is advocated to achieve the maximum disinfection along with antimicrobial irrigants such as sodium hypochlorite in multirooted teeth [7].

In vitro investigations [8,9,10] examined the antibacterial efficacy of sodium hypochlorite gel and aqueous solution and found it to be similar for both forms. However, there are no clinical trials to support this. Hence, it is expected that using sodium hypochlorite gel can reduce the apical extrusion of debris and decrease its side effects. In addition, if the gel and solution forms are equality effective, the benefits of the gel form in root canal treatment cannot be overlooked.

Accordingly, if the gel and aqueous solution forms are found to be equally effective in clinical scenarios, sodium hypochlorite accidents can be reduced and the same concentration can be utilized for the efficient disinfection of perforation cases, young individuals with teeth with open apices, and teeth with root resorption, etc.

On the basis of the above background, the aim of the study was the comparative evaluation of the antibacterial efficacy of 5.25% sodium hypochlorite gel and aqueous solution as a root canal disinfectant in multirooted teeth with primary endodontic lesions. The null hypothesis stated that there would be no difference between the antibacterial efficacy of the 5.25% sodium hypochlorite gel and the aqueous solution as a root canal disinfectant in multirooted teeth with primary endodontic lesions.

## 2. Materials and Methods

**Figure 1:**
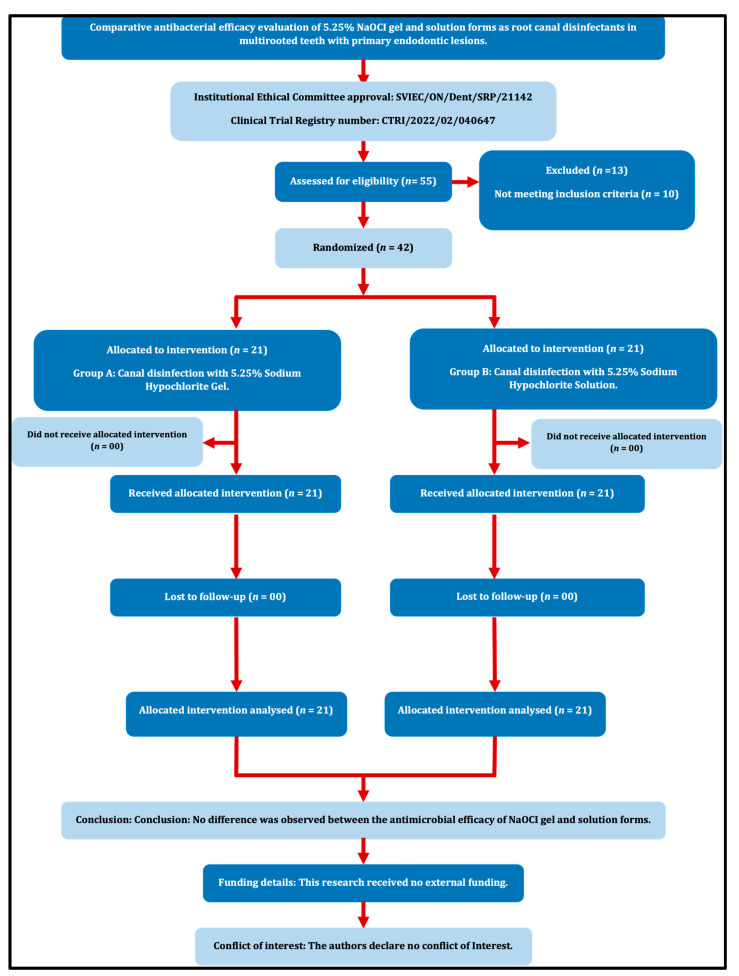
PRIRATE 2020 Flow diagram.

Prior ethical approval was received from the Institutional Ethics Committee (SVIEC/ON/Dent/SRP/21142). The present study followed the PRIRATE guidelines 2020 (Figure 1), and the protocols were registered at www.ctri.nic.in (CTRI/2022/02/040647). The sample size was estimated based on a study conducted by Abu Hasna et al. [11] and a minimum of 42 patients (21 per group) are required for the present study to estimate the mean difference in Colony-Forming Units (CFUs) between groups as 15 × 10^4^ with SD 17 × 10^4^ at 5% alpha error, 95% confidence, and 80% power. No dropouts were anticipated because pre- and post-treatment samples were collected during the same visit.

Each patient provided written informed consent after being briefed about the study and its potential consequences. Patients between 18 and 60 years of age who were systemically healthy Class I patients according to the American Society of Anaesthesiology [ASA] classification [12], with multirooted teeth diagnosed with pulpal necrosis and asymptomatic apical periodontitis with at least three intact coronal walls indicated for endodontic therapy were included for the study.

Patients with root resorption, calcification, developmental anomalies, symptomatic reversible or irreversible pulpitis, acute apical abscess, phoenix abscess, weeping canals, periapical cyst, vertical root/crown fracture, root caries or severe dilacerations, or who were on antibiotic therapy, were excluded from the study. Patients who did not agree to sign the consent form and pregnant females were also excluded from the study.

### 2.1. Clinical Protocol

The clinical procedure was started by performing the dental prophylaxis of the patients with poor oral hygiene and the administration of 2% lignocaine hydrochloride with 1:200,000 adrenaline (Aquacaine plus, Mumbai, India). Following which caries excavation and the straight-line access cavity was prepared with sterile Endo access (Dentsply Sirona, Charlotte, NC, USA) and Endo Z (Dentsply Sirona, USA) burs under rubber dam isolation (Hu-Friedy, Frankfurt, Germany) which was disinfected with 2.5% sodium hypochlorite (Cerkamed, Stalowa Wola, Poland). If a Class II access cavity was present, after total caries excavation and access opening, pre-endodontic build-up was carried out with composite resin (3M ESPE Filtek Z350XT, Maplewood, MN, USA) before the application of a rubber dam. Dam seal material (Prevest Gingiva Shield VLC, Jammu, India) was used in case of any leakage. The canal orifice location was carried out with DG-16 explorer (Hu-Friedy, Rockwell, Chicago, IL, USA), followed by glide path establishment with no.10 K file (Mani, INC, Utsunomiya, Tochigi, Japan) and pulp extirpation with sterile broach (Mani, INC, Utsunomiya, Tochigi, Japan), without using any irrigant. Using an electronic apex locator (Root X mini-J Morita, CA, USA), working length was calculated. An intraoral periapical radiograph was then used to confirm the results.

### 2.2. Pre-Operative Sample Collection

The largest canal in each of the teeth included (Distal canal for mandibular molars and Palatal canal for maxillary molars) was humidified with 5 mL of sterile saline solution for pre-operative sample (S1) collection, and a microbiological sample was obtained by inserting sterile paper points (Hygenic, Coltene) into the canal 1 mm shorter than working length such that they remained in the canal for 60 s with pumping movements to generate a suspension with bacteria and were immediately transferred to a sterile microtube with 2 mL of sterile saline solution. 

### 2.3. Randomization and Allocation 

Before chemo-mechanical preparation, the teeth were divided into two groups at random using the computer randomization method (www.randomizer.org, Accessed on 31 January 2022)

Group A: Canal disinfection with 5.25% sodium hypochlorite gel (N = 21) 

Group B: Canal disinfection with 5.25% sodium hypochlorite aqueous solution (N = 21)

Allocation concealment was accomplished utilizing the SNOSE (Sequentially Numbered Opaque Sealed Envelope) method with a 1:1 allocation ratio. The patients and microbiologist were blinded throughout the study (Double Blinded Study).

### 2.4. Root Canal Disinfection

#### 2.4.1. Group A–Canal Disinfection with 5.25% Sodium Hypochlorite Gel (N = 21)

Depending on the canal shape and variations, chemo-mechanical preparation was accomplished using a step-back, crown-down, or hybrid technique at the full working length. Between each instrumentation motion, 5.25% sodium hypochlorite gel (Chloraxid, Cerkamed, Stalowa Wola, Poland) was injected directly into the canal using the applicator supplied by the manufacturer. Root canal preparation was then completed. Before final irrigation, the canal was filled with 5.25% sodium hypochlorite gel (Chloraxid, Cerkamed, Stalowa Wola, Poland) to which 2 mL of saline was added and Passive Ultrasonic Irrigation [11] (Aceton Satelec P5 Booster scaler, Saint Neots, UK) was carried out with an Irrisafe Passive Ultrasonic Irrigation file (Acteon Satelec, Saint Neots, UK) for 1 min. 

#### 2.4.2. Group B–Canal Disinfection with 5.25% Sodium Hypochlorite Aqueous Solution (N = 21)

Between each instrumentation motion, root canal irrigation was carried out with 1 mL of 5.25% sodium hypochlorite aqueous solution (Chloraxid, Cerkamed, Stalowa Wola, Poland) using a side-vented irrigation needle (Prime Dental RC Twents, Thane, Maharashtra, India). Before final irrigation, the canal was filled with 5.25% sodium hypochlorite aqueous solution (Chloraxid, Cerkamed, Stalowa Wola, Poland) and Passive Ultrasonic Irrigation (Aceton Satelec P5 Booster scaler, Saint Neots, UK) was carried out with an Irrisafe Passive Ultrasonic Irrigation file (Aceton Satelec, Saint Neots, UK) for 1 min. Final irrigation for both the groups was carried out with normal saline using a side-vented irrigation needle (Prime Dental RC Twents, Thane, Maharashtra, India). 

### 2.5. Post-Operative Sample Collection

A post-operative (S2) sample was taken from the same canal with a sterilized paper point in a similar manner as stated for S1 and immediately transferred to a sterile microtube with 2 mL of sterile saline solution. After post-operative sample collection, the teeth allocated to the 5.25% sodium hypochlorite gel group were irrigated again with the 5.25% sodium hypochlorite aqueous solution using standard protocols, followed by irrigation with sterile saline solution. 

After post-operative sample collection, Calcium hydroxide (RC Cal, Prime Dental, Thane, Maharashtra, India India) was used as an intracanal medicament in the canals and the access cavities were temporarily restored using Cavit (3M ESPE, Maplewood, MN, USA).

### 2.6. Microbiological Analysis

All the paper points (S1 and S2) from both the groups were placed on BHI agar plates (TM Media, New Delhi, India) that were incubated aerobically for 48 h and the colony-forming units (CFUs) were then calculated. The colony-forming units in the S1 sample were regarded as the pre-operative microbial load and colony-forming units from S2 sample were considered as the post-operative microbial load. After 48 h, the growth on the culture plates (Central Drug House fine chemical, New Delhi, India) was assessed and the total colony-forming units were recorded using a colony counter (SKY Technology, Panchkula, India).

(1) $$COLONY-FORMING\;UNITS=\frac{Number\;of\;colonies\times Dilution\;factor}{Volume\;Inoculated}$$

**Figure 2:**
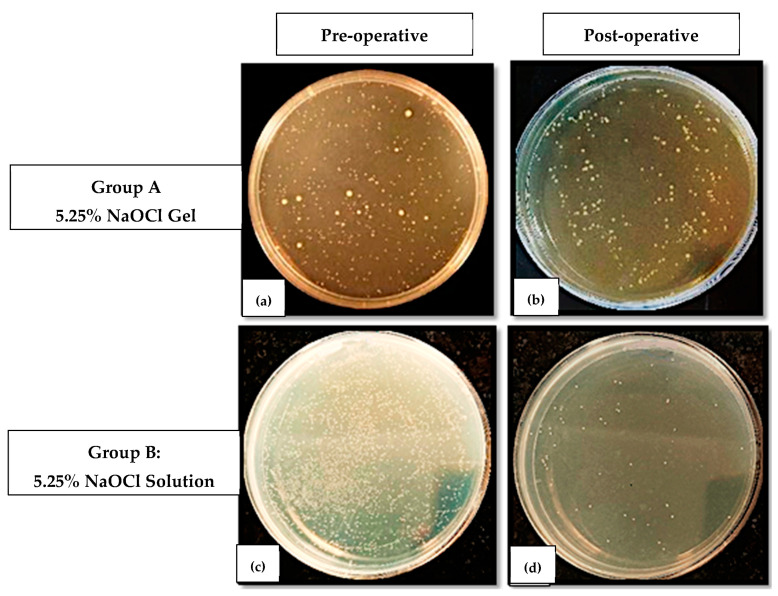
Colony-forming units on BHI agar plates in (**a**,**b**) Pre-operative (S1) and Post-operative (S2) samples for Group A; (**c**,**d**) Pre-operative (S1) and Post-operative (S2) samples for Group B.

This colony-forming units per mL (×10^5^) value was considered as the microbial load of the respective samples (S1 or S2) for both the groups. The difference between the colony-forming units per mL (×10^5^) values of S1 and S2 in a group was considered as the reduction in microbial load after root canal disinfection and this value was considered for comparison of the antimicrobial efficacy of both groups (Figure 2).

### 2.7. Statistical Analysis

This was carried out using SPSS 20.0 software (IBM SPSS Statistics, Chicago, IL, USA). The Shapiro–Wilk test and the Lilliefors Significance Correction were used to test the null hypothesis that the data come from a normally distributed population and the Mann–Whitney U test was used to compare the CFU difference (×10^5^) of the two groups. A paired t-test was used to compare the before and after values in each group, indicating the reduction in the colony-forming units’ count as the colony-forming units difference in each group (intra-group analysis). 

## 3. Results

The present clinical study aims to evaluate and compare the antimicrobial efficacy of 5.25% sodium hypochlorite gel and aqueous solution as a root canal disinfectant in multirooted teeth with primary endodontic lesions.

Out of 42 patients, 21 patients were allocated to each of the groups A and B, where 9 and 13 patients were males and 12 and 8 were females in Groups A and B, respectively. In Group A, 7 patients belonged to the age group of 18–40 years and 14 belonged to the age group of 40–60 years, while in Group B, 11 patients belonged to the age group of 18–40 years and 10 belonged to age group of 40–60 years. In Group A, out of 21 teeth, 11 were maxillary and 10 were mandibular teeth, while in Group B, out of 21 teeth, 12 were maxillary and 9 were mandibular teeth. There were 4 premolar and 17 molar teeth in Group A, while 3 premolar and 18 molar teeth were included as samples in Group B.

On intra-group analysis, there was a highly statistically significant difference (*p* < 0.001) found in the colony-forming units in the S1 (Pre-operative) sample (×10^5^) values and the colony-forming units in the S2 (Post-operative) sample (×10^5^) values in both Groups A and B, analyzed using a paired t-test, showing that there was a significant reduction in the colony-forming units after disinfection in both the groups, and both the disinfectants (5.25% sodium Hypochlorite gel and solution) were highly capable of reducing the bacterial load at the end of chemo-mechanical preparation (Table 1).

The normality test was performed using the Shapiro–Wilk test and the Lilliefors Significance Correction to test the null hypothesis that data come from a normally distributed population. The Shapiro–Wilk test showed that the CFU in S1 Sample (×10^5^), the CFU in S2 sample (×10^5^), and the CFU difference (×10^5^) all show statistically significant values (<0.05), indicating that the data is skewed in distribution and is non-parametric in nature (Table 2). Therefore, a non-parametric test should be used for comparison of the CFU difference (×10^5^) between Group A and Group B. Hence, the Mann–Whitney test was used for intergroup comparison.

Comparison of the colony-forming units difference (×10^5^) between the two groups (Table 3), showed that the colony-forming units difference (×10^5^) was higher in Group B (2 ± 1.82) (5.25% sodium hypochlorite solution group) as compared to Group A (1.94 ± 1.36) (5.25% sodium hypochlorite gel group), showing that there was a greater reduction in the bacterial load when the canals were disinfected using 5.25% sodium hypochlorite solution as compared to 5.25% sodium hypochlorite gel. However, there was no statistically significant difference between the two groups when the Mann–Whitney U test was used to compare the CFU difference (×10^5^) of the two groups (*p* = 0.744).

## 4. Discussion

Bacteria and their by-products are the leading causes of pulpal and periapical diseases, along with the failure of the endodontic treatment [13,14]. The techniques, instruments, and irrigants currently available limit root canal sterilization. Furthermore, the goal of chemo-mechanical preparation, which includes both mechanical instrumentation and chemical irrigation, should be to reduce intra-canal bacterial populations to levels compatible with periapical tissue healing [15], because bacteria that remain in the root canal after root filling are responsible for persistent infections and treatment failures [16].

It is necessary to use an ideal intracanal irrigant that eliminates microorganisms, dissolves necrotic tissue, and does not irritate healthy tissues. Owing to its wide-spectrum antimicrobial activity, tissue-dissolving properties, and lubrication properties, sodium hypochlorite is the irrigant that is most frequently employed during endodontic therapy [17,18,19]. In the endodontic literature, sodium hypochlorite values ranging from 0.5% to 5.25% were reported as adequate for antimicrobial action. Although 2.5% is the concentration that is most frequently employed, greater concentrations (5.25%) of sodium hypochlorite have stronger antimicrobial effects and tissue-dissolving abilities [20]. Furthermore, when Berber et al. conducted a study to compare the efficacy of various concentrations of sodium hypochlorite and instrumentation techniques in reducing E. faecalis within root canals and dentinal tubules, it was discovered that 5.25% NaOCl was the most efficient irrigant solution followed by 2.5% NaOCl, at all thirds and depths of root canals and for all the instrumentation techniques [21]. Hence, a 5.25% concentration of sodium hypochlorite in two different forms (gel and aqueous solution) was used for disinfection of root canals in the present study.

Nonetheless, because of various complications such as hemolysis, necrosis, inflammatory response, and tissue destruction, many dentists avoid using sodium hypochlorite frequently in daily practice, particularly in cases with open/wide apex, perforations, or resorption because they are afraid it would protrude over the apex [22].

Since sodium hypochlorite gel has less apical extrusion than sodium hypochlorite solution and theoretically has the same antimicrobial effect, it has been proposed as an alternative endodontic irrigant [23]. Controlling its viscosity was necessary to prevent it from extruding past the apex, thus avoiding such clinical complications, and the gel form of sodium hypochlorite was introduced to achieve this. The gel form of sodium hypochlorite is formed by the process of gelation, where gelators are added to a hot solution of sodium hypochlorite. These gelators tend to self-assemble by formation of H-bonds but they are quickly broken due to high temperature; however they are re-established on cooling thus giving a 3-dimensional network of gel which has higher viscosity, thus solving the problem of extrusion beyond the apex [24].

Passive ultrasonic irrigation generates vibration that creates an acoustic stream that creates a shear stress to dislocate debris inside instrumented root canals, increasing the irrigant’s penetration into dentinal tubules [9]. In order to improve the elimination of the smear layer and the entry of liquid into the apical third, ultrasonic vibrations travel inside the irrigant solution to form micro-cavitations that implode [25].

Passive ultrasonic irrigation has been demonstrated by Mohmmed et al. to be efficient in removing biofilm from lateral canals [26]. So, in the present study and following chemo-mechanical preparation, passive ultrasonic irrigation was applied to both the groups for an equal amount of time, to enhance the smear layer removal and ensure admission of the disinfectant into the apical third of the root canals.

In an in vitro experiment, Shamsi et al. [8] compared the antibacterial effects of sodium hypochlorite gel and solution on Enterococcus faecalis (*E. faecalis*), and they found that both the solution and gel of sodium hypochlorite 5.25% were equally effective. This outcome was consistent with research by Hasna et al. [11] and Luz et al. [27], who found that although the tissue dissolution capacity of the sodium hypochlorite solution was higher than that of the gel form, both the sodium hypochlorite gel and the solution were effective in reducing the microbial load of *E. coli* and *E. faecalis* after treatment at different concentrations. Furthermore, in an in vivo study conducted by Karatas et al., using sodium hypochlorite gel during root canal preparation resulted in less post-operative pain on day 1 when compared to sodium hypochlorite solution [28]. 

However, due to the increase in viscosity its penetration into dentinal tubules is limited, as indicated by an in vitro study conducted by Faria et al. where, when compared to the gel form, the 3% sodium hypochlorite solution penetrated dentinal tubules significantly better (*p* < 0.05) [9]. This can be regarded as a constraint of the gel form of sodium hypochlorite, along with other issues such as questionable penetration into inaccessible areas, failure to flush out debris from root canals, and the requirement of adding saline solution along with the gel for passive ultrasonic irrigation, which may cause the sodium hypochlorite gel to become diluted and to lose its effectiveness.

To the best of our best knowledge, there are no clinical studies comparing the antimicrobial efficacy of 5.25% sodium hypochlorite aqueous solution and 5.25% sodium hypochlorite gel, and hence this study was conducted as a clinical study will provide a better understanding of the antimicrobial efficacy of the two root canal disinfectants. 

In the present study, the colony-forming units difference (×10^5^) in S1 and S2 samples is higher in Group B (5.25% sodium hypochlorite aqueous solution) as compared to Group A (5.25% sodium hypochlorite gel), showing that the antimicrobial efficacy of the solution form was greater than the gel form. However it was not statistically significant (*p* value = 0.744) and this result was consistent with the findings of studies conducted by Shamsi et al. [8], Hasna et al. [11] and Luz et al. [27]. On the contrary, these findings were in contrast with the findings of studies conducted by Poggio et al. [29] and Zand et al. [30]. It is important to note that all of these were in vitro studies; however this is an in-vivo study that is a better scenario for assessing the clinical antimicrobial efficacy of the disinfectants used. 

Therefore, the null hypothesis of the study that there will be no difference in the antimicrobial efficacy of 5.25% sodium hypochlorite gel and 5.25% aqueous solution when employed as root canal disinfectants was not rejected. 

The reason for the similar antimicrobial efficacy of both the groups, 5.25% sodium hypochlorite gel and 5.25% sodium hypochlorite aqueous solution, may be the fact that a similar concentration of the irrigant was used and the samples were collected from the largest canals with least amount of anatomical variations expected, showing that both forms of sodium Hypochlorite are equally effective in eliminating microbes from such canals. Moreover, Passive Ultrasonic Irrigation was employed for the groups for an equal amount of time, something which would have assisted in achieving effective disinfection for both the groups. The reason for the lower, although not statistically significant, antimicrobial efficacy of sodium hypochlorite gel in some samples may be that saline solution needs to be added along with the gel for passive ultrasonic irrigation, which might result in the dilution of the sodium hypochlorite gel and, in turn, reduce its effectiveness.

In the present study, only 5.25% sodium hypochlorite gel was used for root canal disinfection. Further studies need to be conducted with other concentrations of sodium hypochlorite gel in order to confirm their antimicrobial efficacy. Moreover, the samples used for the current investigation were taken from the largest canal with the fewest variances expected, which may be the cause of the groups’ identical antimicrobial efficacy results, hence further studies need to be carried out where samples are taken from more difficult canals. The root canal is home to a varied flora, however only the aerobic culture method was used to quantify the residue, hence more studies need to be conducted where other all kinds of microflora are detected. The sample size taken in this study is relatively small, hence more studies with larger sample sizes should be conducted to quantify the antimicrobial efficacy of sodium hypochlorite gel.

## 5. Conclusions

Within the limitations of the study, it could be concluded that 5.25% sodium hypochlorite gel has similar antimicrobial efficacy as the solution form, and it can be used for effective disinfection in cases with wide/open apex, perforation, or resorption. However it is still questionable if it can replace 5.25% sodium hypochlorite solution.

## Figures and Tables

**Table 1:** Comparison of the before and after values with respect to the colony-forming units.

		N	Mean ± SD	Mean Difference ± SD	t	*p* Value
Group A (Gel)	Colony-Forming Units in S1 Sample (×10^5^)	21	2.19 ± 1.31	1.94 ± 1.36	6.53	<0.001 *
	Colony-Forming Units in S2 sample (×10^5^)	21	0.25 ± 0.47			
Group B 5.25% (Aqueous solution)	Colony-Forming Units in S1 Sample (×10^5^)	21	2.29 ± 1.77	2 ± 1.82	5.03	<0.001 *
	Colony-Forming Units in S2 sample (×10^5^)	21	0.29 ± 0.32			

* Statistically significant value after the application of paired *t*-test. S1-pre-operative sample; and S2-post-operative sample.

**Table 2:** Tests of Normality using Shapiro–Wilk test and Lilliefors Significance Correction.

	Group	Shapiro–Wilk		
		Statistic	df	Sig.
CFU in S1 Sample (×10^5^)	Group A (Gel)	0.825	21	0.002 *
	Group B (Aqueous solution)	0.843	21	0.003 *
CFU in S2 sample (×10^5^)	Group A (Gel)	0.547	21	0.000 *
	Group B (Aqueous solution)	0.800	21	0.001 *
CFU difference (×10^5^)	Group A (Gel)	0.815	21	0.001 *
	Group B (Aqueous solution)	0.842	21	0.003 *

* Statistically significant value. Colony-forming Units; S1-pre-operative sample; and S2-post-operative sample.

**Table 3:** Inter-group analysis using Mann–Whitney U test.

	Group	N	Mean ± SD	Mean Rank	Sum of Ranks	Mann–Whitney U	*p*-Value
CFU in S1 Sample (×10^5^)	Group A (Gel)	21	2.19 ± 1.31	21.93	460.50	211.500	0.821
	Group B (Aqueous solution)	21	2.29 ± 1.77	21.07	442.50		
	Total	42					
CFU in S2 sample (×10^5^)	Group A (Gel)	21	0.25 ± 0.47	18.76	394.00	163.000	0.148
	Group B (Aqueous solution)	21	0.29 ± 0.32	24.24	509.00		
	Total	42					
CFU difference (×10^5^)	Group A (Gel)	21	1.94 ± 1.36	22.12	464.50	207.500	0.744
	Group B (Aqueous solution)	21	2.01 ± 1.82	20.88	438.50		
	Total	42					

CFU—Colony-forming Units; S1-pre-operative sample; and S2-post-operative sample.

## Data Availability

The present study protocol is registered with www.ctri.nic.in (CTRI/2022/02/040647).
